# Bizarre Parosteal Osteochondromatous Proliferation (BPOP) of the Hand

**DOI:** 10.5334/jbsr.4138

**Published:** 2026-01-27

**Authors:** Dheysel Tenzin, Pieter Van Geel, Jonas De Melio

**Affiliations:** 1 Resident in Radiology, Ghent University, Belgium; 2 Orthopaedic Surgeon, AZ Sint Blasius Dendermonde, Belgium; 3 Radiologist, AZ Sint Blasius Dendermonde, Belgium

**Keywords:** BPOP, hand, bizarre parosteal osteochondromatous proliferation, bone lesion

## Abstract

Bizarre parosteal osteochondromatous proliferation (BPOP), or Nora lesion, is a rare benign condition that can mimic other pathologies. A case is described of a 66-year-old man with a slowly enlarging nodule on his finger over five years. Imaging showed a parosteal lesion without continuity with the trabecular bone, strongly suggestive of BPOP. The diagnosis was histologically confirmed. This case highlights the importance of recognizing the characteristic imaging features of BPOP.

*Teaching point:* BPOP may appear as a worrisome lesion on imaging, but its typical location and lack of continuity with the trabecular bone should raise awareness for BPOP.

## Introduction

Bizarre parosteal osteochondromatous proliferation (BPOP), also known as Nora lesion, is a rare benign exophytic osteochondral lesion typically seen in the small bones of the hands and feet. It was first described in 1983 by Nora et al. [1]. It may present with a rapid growth and an aggressive radiological appearance could raise suspicion for malignancy. While histopathology remains the gold standard for the diagnosis, radiological imaging has a role in the initial detection and narrowing the differential diagnosis. This case emphasizes the characteristic imaging features across multiple modalities and their role in distinguishing BPOP from other surfacing bone lesions.

## Case Report

A 66-year-old man presented to the radiology department with a gradually enlarging, hard nodule on the radial side of the right index finger, present for approximately five years. Past medical history only showed meningioma resection.

**Figure 1:**
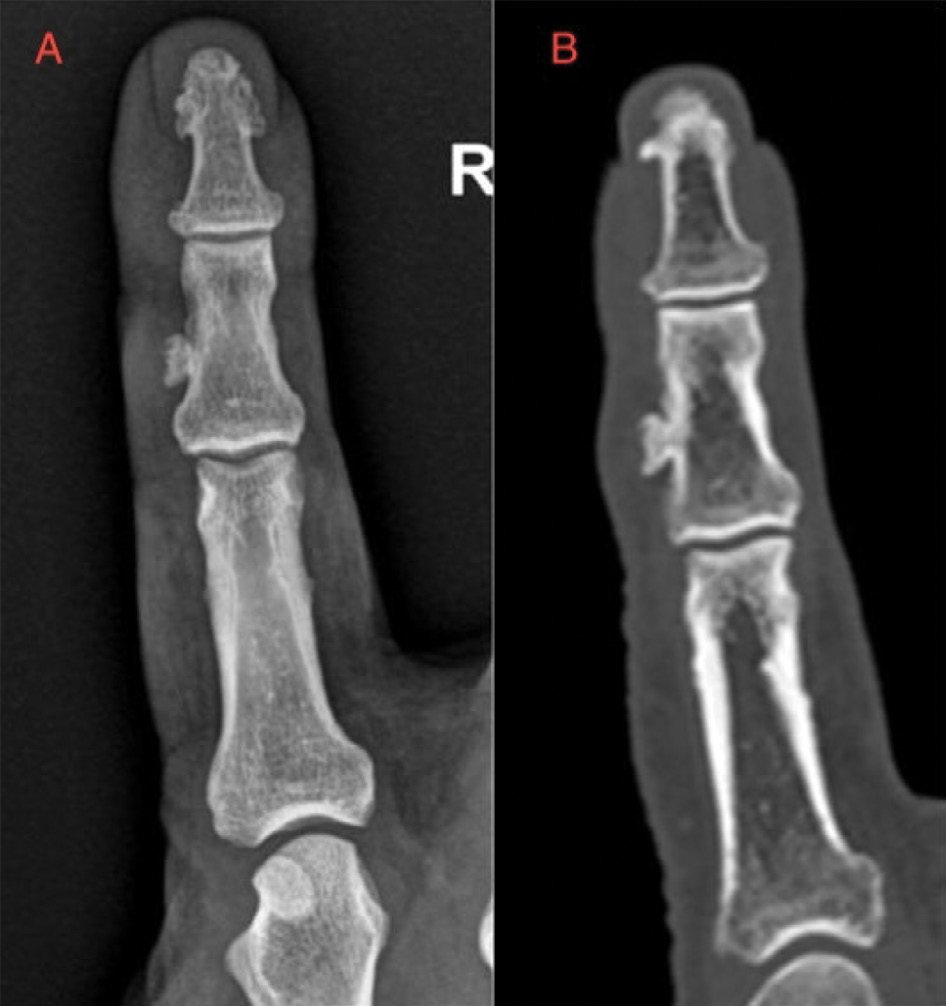
Lesion on radiography **(A)** and on computed tomography **(B)**.

A conventional X-ray and ultrasound of the right index finger showed a 6 mm parosteal bony structure along the radial side of the middle phalanx of the right index finger. No clear continuity with the trabecular bone was seen, raising suspicion for a BPOP (Figure 1A).

To further assess the lesion a computed tomography (CT) scan of the hand was performed. This confirmed a parosteal lesion with cortical and trabecular components but lacked continuity with the underlying trabecular bone of the middle phalanx. The superficial part of the lesion showed irregularity, suggesting a thin cartilaginous cap (Figure 1B). There was no periosteal reaction.

**Figure 2:**
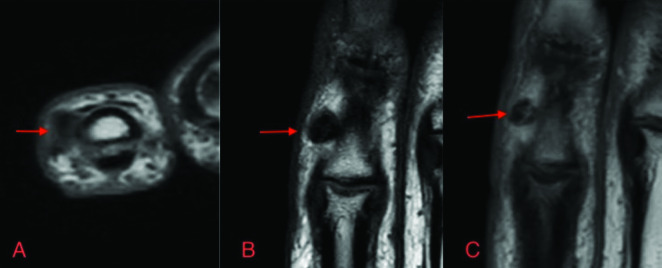
MRI of the lesion. Central hyperintensity on axial T2 **(A)**. Peripheral and central hypointensity on T1 **(B)**. Discrete central contrast enhancement on T1 **(C)**.

Magnetic resonance imaging (MRI) of the hand which showed no continuity of the trabecular bone or cartilaginous cap. The signal characteristics of the lesion showed peripheral T1- and T2-hypointense signal and central T1 hypointense and T2 heterogeneous to hyperintense signal. There was mild contrast enhancement in the lesion (Figure 2).

**Figure 3:**
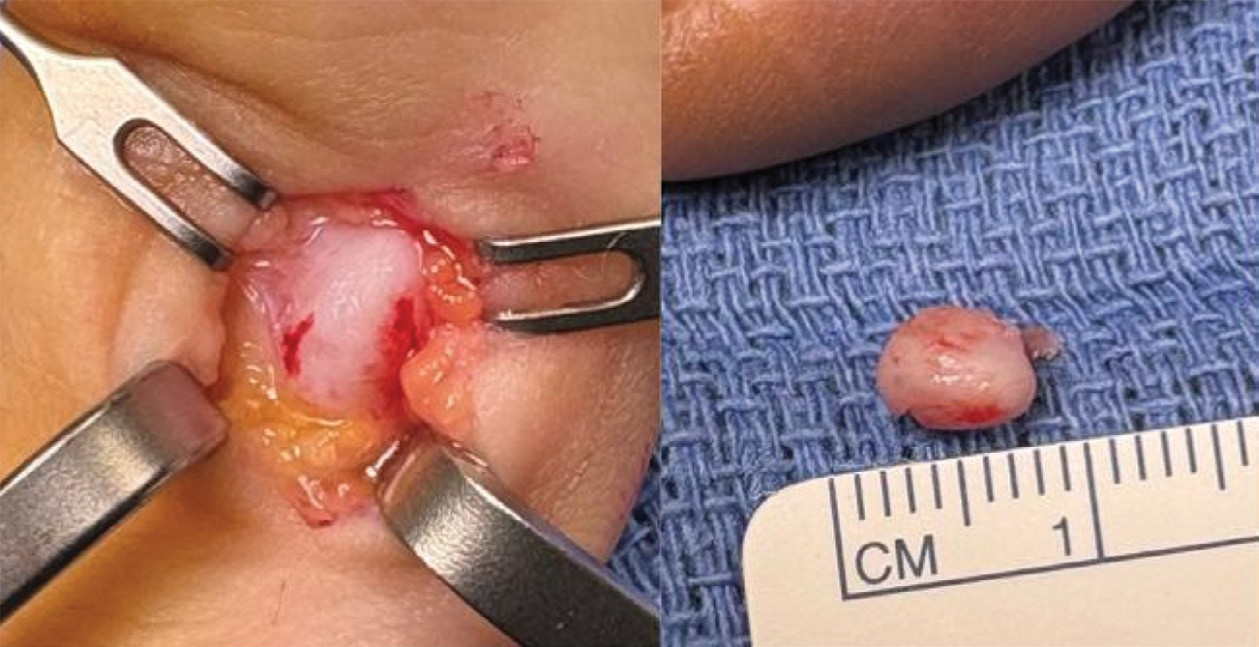
Resection of the lesion measuring roughly 5 mm.

Surgical resection of the lesion followed (Figure 3). Histopathological analysis described areas of slight ‘blue bone,’ consistent with BPOP. Malignancy was excluded.

## Discussion

BPOP is a rare benign lesion, mostly seen in adults between the 20s and 40s but can also occur at older ages. The typical location is in the hands and feet. Despite being benign, BPOP may display features that mimic malignancy, due to potential rapid growth and irregular ossification.

Typical radiological imaging findings are a well-demarcated bony structure attached to the cortex and lacking continuity of the trabecular bone. This is almost pathognomonic for BPOP.

CT and MRI imaging can further characterize the trabecular and cortical components. CT shows the same imaging features as radiography. MRI can help to exclude marrow involvement and other signs of malignancy. BPOP typically has a low signal on T1-imaging and an intermediate signal on T2-imaging. In most cases, there is a normal cortical appearance and absence of marrow signal changes [2].

Histology shows a typical mix of cartilage, bone, and fibrous tissue in a disorganized architecture. The presence of the ‘blue cells’ contributes to the diagnosis of BPOP. Blue cells are immature chondrocytes in the cartilaginous component of the lesion that appear basophilic or blue on hematoxylin and eosin (H&E) stain. Absence of malignant signs, such as infiltrative permeation into surrounding structures, severe nuclear atypia, and necrosis, is essential.

The differential diagnosis includes osteochondroma, myositis ossificans, periosteal chondroma, and osteosarcoma. The key characteristic with a lack of trabecular bone continuity and cortical origin can help differentiate these entities.

BPOP has a risk of recurrence of 50% after surgical resection [3]. Therefore, long-term follow-up is recommended.

## Conclusion

This case report illustrates a case of BPOP with classic radiological features and the importance to include this in the differential diagnosis for slow-growing, cortical-based lesions of the hand and feet. Since it is a benign and rare lesion with typical radiological features, awareness of this entity amongst radiologists is raised to avoid prompt misdiagnosis as a malignant lesion.
